# International Variation in Criteria for Internal Mammary Chain Radiotherapy

**DOI:** 10.1016/j.clon.2019.04.007

**Published:** 2019-07

**Authors:** F.K. Duane, P. McGale, S. Teoh, C. Mortimer, J. Broggio, S.C. Darby, D. Dodwell, B. Lavery, S. Oliveros, K.A. Vallis, C.W. Taylor

**Affiliations:** ∗ St Luke's Radiation Oncology Network, St. James's Hospital, Dublin, Ireland; † Clinical Trial Service Unit, Nuffield Department of Population Health, University of Oxford, Oxford, UK; ‡ CRUK/MRC Oxford Institute for Radiation Oncology, Department of Oncology, University of Oxford, Oxford, UK; § Public Health England, Birmingham, UK; ¶ Oxford University Hospitals NHS Foundation Trust, Oxford, UK

**Keywords:** Internal mammary chain, international criteria, radiotherapy

## Abstract

**Aims:**

Evidence has emerged that internal mammary chain (IMC) radiotherapy reduces breast cancer mortality, leading to changes in treatment guidelines. This study investigated current IMC radiotherapy criteria and the percentages of patients irradiated for breast cancer in England who fulfilled them.

**Materials and methods:**

A systematic search was undertaken for national guidelines published in English during 2013–2018 presenting criteria for ‘consideration of’ or ‘recommendation for’ IMC radiotherapy. Patient and tumour variables were collected for patients who received breast cancer radiotherapy in England during 2012–2016. The percentages of patients fulfilling criteria stipulated in each set of guidelines were calculated.

**Results:**

In total, 111 729 women were recorded as receiving adjuvant breast cancer radiotherapy in England during 2012–2016 and full data were available on 48 095 of them. Percentages of patients fulfilling IMC radiotherapy criteria in various national guidelines were: UK Royal College of Radiologists 13% (6035/48 095), UK National Institute for Health and Care Excellence 18% (8816/48 095), Germany 32% (15 646/48 095), Ireland 56% (26 846/48 095) and USA 59% (28 373/48 095). Differences between countries occurred because in Ireland and the USA, treatment may be considered in some node-negative patients, whereas in the UK, treatment is considered if at least four axillary nodes are involved or for high-risk patients with one to three positive nodes. In Germany, treatment may be considered for all node-positive patients.

**Conclusions:**

There is substantial variability between countries in criteria for consideration of IMC radiotherapy, despite guidelines being based on the same evidence. This will probably lead to large variations in practice and resource needs worldwide.

## Highlights

• Criteria for consideration of IMC RT vary substantially between countries.
• IMC RT may be considered for ∼13–18% in the UK (high-risk N+ disease).
• A third of patients for breast cancer radiotherapy have node-positive disease.
• IMC RT may be considered for up to 60% in the US (includes high-risk N0 disease).

## Introduction

Breast cancer radiotherapy reduces the risks of cancer recurrence and death in patients irradiated after breast-conserving surgery and after mastectomy for node-positive disease [1], [2]. In breast cancer, there are a number of different target regions that may be irradiated. Meta-analyses of randomised trials have shown that if radiotherapy is given after breast-conserving surgery it should include the conserved breast and if given after mastectomy it should include the chest wall. For patients undergoing nodal radiotherapy there has been debate concerning the role of internal mammary chain (IMC) radiotherapy. This has been reflected in discrepancies in country-specific guidelines and practice [3], [4], [5], [6], [7], [8], [9], [10], [11], [12], [13]. Even within some countries, breast cancer radiotherapy practice has varied substantially [14].

In 2015, two large randomised trials including about 6000 patients showed that irradiating the IMC significantly reduced breast cancer mortality [15], [16]. A large cohort study including about 3000 patients, in which IMC radiotherapy was allocated according to tumour laterality, also provided support for IMC irradiation [17]. In countries where IMC radiotherapy has been used routinely in node-positive breast cancer, these findings will have little impact on practice [18]. In other countries, these publications have changed radiotherapy guidelines and will affect practice appreciably [9], [10], [13]. In the UK, IMC radiotherapy was previously recommended only in patients with known IMC metastases [3]. This changed in 2016, when a consensus statement was published by the Royal College of Radiologists (RCR). The consensus was that IMC radiotherapy should be considered in patients with T4 (extension to the chest wall or skin) and/or N2–3 (metastases in greater than three axillary nodes, fixed/matted axillary nodes or in internal mammary, supraclavicular or infraclavicular nodes) disease and in patients with medial or central breast cancers and one to three axillary macrometastases (>2 mm in size) who have been recommended locoregional irradiation based on other risk factors [19], [20]. In 2018, the National Institute for Health and Care Excellence (NICE) published updated clinical practice guidelines [4] that included a new statement that IMC radiotherapy should be considered for all patients with node-positive disease (macrometastases) who were planned to receive locoregional irradiation. This would include patients with greater than three axillary nodes and others with one to three positive axillary nodes, and with other poor prognostic factors. Such changes in national recommendations over recent years will affect the number of patients receiving IMC radiotherapy, which affects radiotherapy planning and contouring and treatment delivery time because IMC radiotherapy can be technically challenging.

This study aims to inform resource allocation in radiotherapy departments using a systematic search for IMC radiotherapy guidelines and data from women who received adjuvant breast cancer radiotherapy in England during 2012–2016. It first investigates the percentage of patients irradiated for breast cancer in England in whom IMC radiotherapy would be considered based on the UK RCR consensus [19] and on UK NICE recommendations [4]. Second, it investigates the percentages of patients who would fulfil criteria for IMC radiotherapy should they receive radiotherapy in other countries.

## Materials and Methods

### Internal Mammary Chain Radiotherapy Guidelines

**Fig 1:**
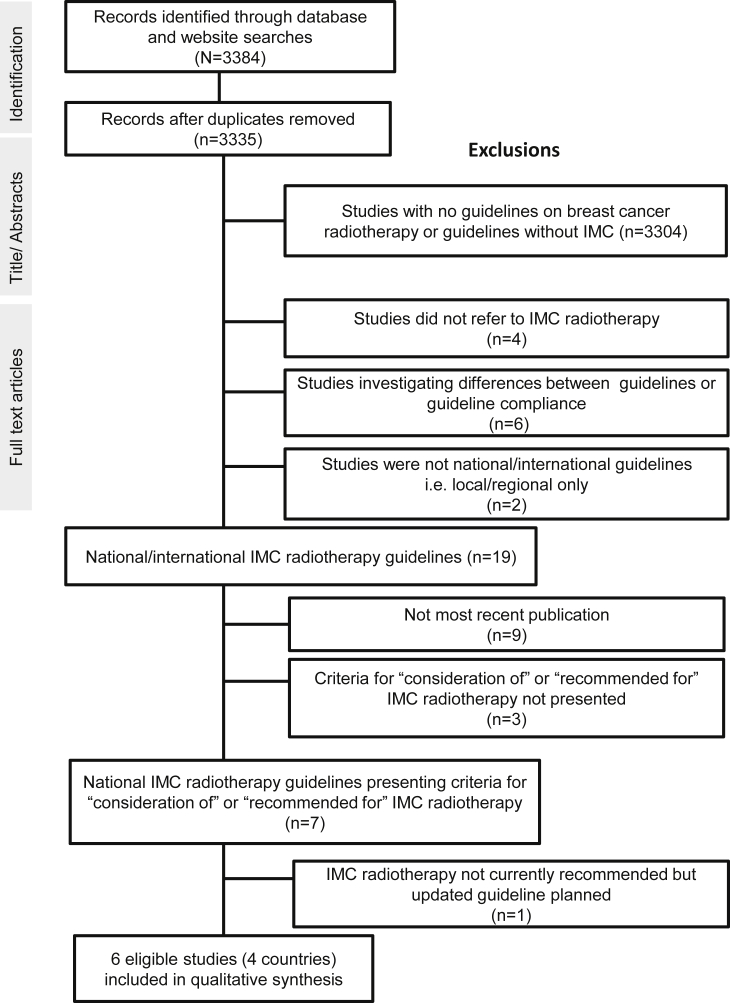
Flowchart for the selected clinical practice guidelines published in English during January 2013 to January 2018.

A systematic search was conducted for country-specific breast cancer radiotherapy guidelines presenting criteria for ‘consideration of’ or ‘recommendation for’ IMC radiotherapy. Studies were identified using the Preferred Reporting Items for Systematic Reviews and Meta-analyses [21] (Figure 1). By the year 2013 the results of two landmark trials investigating the benefit of nodal radiotherapy had been presented at international meetings. The MA20 trial was presented at the American Society for Clinical Oncology Annual Meeting [22] and the European Organization for Research and Treatment of Cancer (EORTC) 22922/10925 trial was presented at the European Cancer Congress [23]. Therefore, the search was limited to studies published between January 2013 and January 2018, as these would probably consider this new evidence. The Medline database was searched for studies published in English using the following terms: ‘breast cancer’ AND ‘radiotherapy’ AND ‘guideline OR statement OR opinion’. Four online guideline databases and 10 websites were also searched using the search term ‘breast cancer’ (Table 1). Local or regional guidelines were excluded. For each guideline, criteria for ‘consideration of’ or ‘recommendation for’ IMC radiotherapy were abstracted and summarised.

**Table 1:** List of electronic databases and websites searched for eligible guidelines∗

Source	Weblink
National Guideline Clearinghouse	www.guideline.gov
National Institute for Health and Care Excellence	www.nice.org.uk
Scottish Intercollegiate Guidelines Network	www.sign.ac.uk
GIN International Guideline Library	www.g-i-n.net/home
European Society for Radiotherapy & Oncology	www.estro.org
National Comprehensive Cancer Network Ireland	www.nccn.org
Canadian Medical Association	www.cma.ca
Royal College of Physicians and Surgeons in Canada	www.royalcollege.ca
Canadian Association of Radiation Oncology	www.caro-acro.ca
Australian Clinical Practice Guidelines	www.clinicalguidelines.gov.au
The Royal Australian and New Zealand College of Radiologists	www.razcr.com
European Society of Medical Oncology	www.esmo.org
Royal College of Radiologists, UK	www.rcr.ac.uk
Ministry of Health, New Zealand	www.health.govt.nz

∗ Accessed 1 September 2018.

### Patients Included, Variables and Information Sources

**Fig 2:**
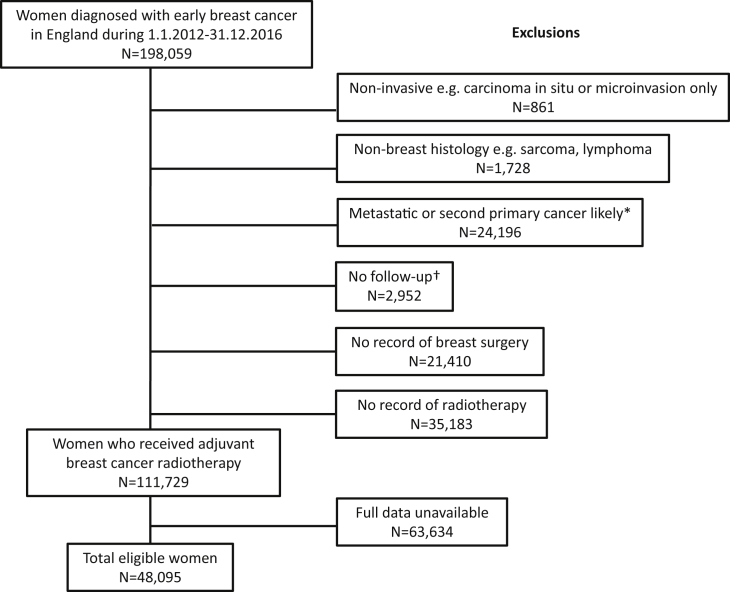
Composition of study population among women diagnosed with breast cancer in England during January 2012 to December 2016. *Women whose treatment intent was probably palliative were identified as follows: record of drug usually given for metastatic disease, record of palliative chemotherapy or palliative radiotherapy within a year of breast cancer diagnosis, record of metastatic disease or second cancer or death within 3 months of breast cancer diagnosis. †Women with no follow-up were excluded because it was not possible to check whether their treatment intent was probably palliative (see above).

Data on all women diagnosed with early breast cancer in the population of England during 2012–2016 were received from Public Health England (Figure 2). These data did not include men with breast cancer but did include the 10% of patients who received neoadjuvant chemotherapy. Data had been linked to several other datasets to identify patients who received treatments for metastatic disease within a year of breast cancer diagnosis or who died, developed metastatic disease or second cancer within 3 months of diagnosis. These women were excluded as their original treatment intent was probably palliative rather than adjuvant (Figure 2). Women were also excluded if they had ductal carcinoma *in situ* or microinvasion alone, if their histology was not breast cancer or if there was no record of breast surgery or of radiotherapy. Patients with available information on the following tumour variables were included in the study: quadrant location, tumour stage, number of positive nodes, oestrogen receptor status, HER2 receptor status and grade (Table 2).

**Table 2:** Patient, tumour and treatment variables among 48,095 women irradiated for early breast cancer in England during 2012–2016

Variable	No. patients	%
**(a) Patient**		
Age at diagnosis (years)		
<40	1730	4
40–49	8114	17
50–59	13 088	27
60–69	15 502	32
70–79	7613	16
80+	2048	4
Geographic region		
East Midlands	5351	11
East of England	6978	15
London	2942	6
North East	3866	8
North West	6151	13
South East	6219	13
South West	6459	13
West Midlands	4200	9
Yorkshire and the Humber	5929	12
**(b) Tumour**		
Breast cancer laterality		
Left	24 602	51
Right	23 477	49
Bilateral/unknown	16	<1
Quadrant location∗		
Lateral	28 191	59
Central	4147	9
Medial	11 448	24
Overlapping	4309	9
Tumour stage∗		
T1: ≤2 cm	29 665	62
T2: >2–5 cm	15 878	33
T3: >5 cm	2284	5
T4: Spread to skin/chest wall	268	1
Number of positive nodes∗		
0	32 449	67
1–3	11 631	24
4–9	2830	6
10 or more	1185	2
Oestrogen receptor status∗		
Positive	42 053	87
Negative	6042	13
HER2 receptor status∗		
Positive	5461	11
Negative	42 634	89
Tumour grade∗		
Low	8627	18
Intermediate	24 950	52
High	14 518	30
**(c) Treatment**		
Type of surgery		
Mastectomy	8617	18
Breast conserving	39 478	82
All women	48 095	100

∗ Information on these tumour factors was necessary for inclusion in the study.

### Patient Categorisation

Each patient was categorised according to whether they met criteria for consideration of IMC radiotherapy (Table 3). For each set of national guidelines, patients in the first row were categorised according to whether they met the criteria. For subsequent rows, any patients who had already been counted in previous rows were omitted so there was no double counting. For example, for the RCR guidelines, a patient with a T4 tumour, one axillary node metastasis, central tumour with risk factors for locoregional radiotherapy scored in row 1 but not in row 2.

**Table 3:** Patients recorded as receiving adjuvant breast cancer radiotherapy in England during 2012–2016 who would fulfil criteria for internal mammary chain radiotherapy in national and international guidelines

Clinical guidelines	Criteria for ‘consideration of’ and ‘recommended for’ internal mammary chain radiotherapy	Patients meeting criteria	%
		Number/48 095	
Royal College of Radiologists, UK (RCR) [19]			
Consider	T4 and/or N2–3 disease	4163	9
Consider	1–3 axillary macrometastases and central/medial disease, who have been recommended		4
	locoregional irradiation based on risk factors∗ (including age and tumour biology)	1872	
		**6035**	**13**
National Institute for Health and Care Excellence (NICE) [4]			
Consider	≥4 positive axillary nodes	4015	8
Consider	1–3 positive nodes if T3/4 or high grade	4801	10
		**8816**	**18**
German Society of Radiation Oncology (DEGRO) [10]†			
Recommend	>3 involved axillary nodes	4015	8
Strongly consider	1–3 involved axillary nodes	11 631	24
		**15 646**	**32**
Department of Health, Ireland (NCCP) [13]			
Consider	Positive axillary nodes	15 646	33
Consider	Negative axillary nodes and inner quadrant tumour	11 200	23
		**26 846**	**56**
National Comprehensive Cancer Network, USA (NCCN) [9]‡§			
Recommend	≥4 positive axillary nodes	4015	8
Strongly consider	1–3 positive axillary nodes	11 631	24
Consider	Negative axillary nodes and tumour >5 cm post-mastectomy	501	1
Consider	Negative axillary nodes, central/medial tumour ≤5 cm post-mastectomy	536	1
Consider	Negative axillary nodes, central/medial tumour post-breast-conserving surgery	10 485	22
Consider	Negative axillary nodes, lateral tumour >2 cm with high-risk features (young age)||	1205	3
		**28 373**	**59**

Table includes six studies in four countries. The UK included two studies [4], [19]. In the USA, a second eligible guideline was identified [12], which was not included in the table. See footnote ‡.

Guidelines are ordered according to the total percentage of women fulfilling criteria.

∗ We used the following risk factors: age ≤50 years, oestrogen receptor negative, HER2 receptor positive and grade 3.

† The DEGRO expert panel also concluded that nodal irradiation can be discussed with node-negative patients with risk factors on a case by case basis.

‡ A second clinical practice guideline (American Society of Clinical Oncology, American Society for Radiation Oncology and Society of Surgical Oncology) [12] from the USA was identified. This guideline only related to radiotherapy for patients who received mastectomy and had T1–2 breast cancer with one to three positive nodes, so it was not included in the table.

§ The NCCN guidelines also reported that internal mammary chain radiotherapy should also be considered for patients post-mastectomy with positive margins when re-excision to negative margins is not feasible. Margin data were unavailable for patients in the listed categories.

|| We defined ≤50 years as young.

The number of patients in whom IMC radiotherapy would be considered or recommended in each set of national guidelines was calculated by totalling the patients who fulfilled each individual criterion (individual rows, Table 3). The percentage of patients in whom IMC radiotherapy would be considered or recommended was calculated by dividing this number by the total number of patients (48 095). Sensitivity analyses were undertaken to assess the impact of missing data (Appendix A).

### Analyses Repeated Using Individual Patient Data in a Single UK Centre

Similar analyses were conducted using data from a review of individual patients' notes for all patients who received adjuvant radiotherapy during a 6-month period in one cancer centre in South East England during 2014 (Appendix B). Percentages of patients who would fulfil criteria for consideration of IMC radiotherapy were calculated and compared with analyses for women in the whole of England (Appendix B).

## Results

### Internal Mammary Chain Radiotherapy Guidelines

Seven publications were identified that presented criteria for ‘consideration of’ or ‘recommendation for’ IMC radiotherapy (Figure 1) [4], [7], [9], [10], [12], [13], [19]. In one of these, IMC radiotherapy was not recommended [7] but the committee who wrote it planned to update the guidance soon, so it was not included. Six eligible studies from four countries presented criteria for ‘consideration of’ or ‘recommendation for’ IMC radiotherapy and were included in the qualitative synthesis (Table 3) [4], [9], [10], [12], [13], [19].

### Included Patients

During the calendar years 2012–2016, 198 059 women in the population of England were diagnosed with early breast cancer (Figure 2); 861 patients were excluded because their pathology was recorded as non-invasive and 1728 because their histology was non-breast, e.g. sarcoma. Another 24 196 patients were excluded because follow-up variables indicated that their original treatment intent may have been palliative rather than adjuvant and 2952 were excluded because no follow-up was available and, therefore, it was not possible to confirm the original treatment intent. In 21 410 patients there was no record of breast surgery and in 35 183 there was no record of radiotherapy. For 63 634 patients, including most of the 12 160 patients who received neoadjuvant chemotherapy, one or more of the essential data items was unavailable. The total number of patients available for analysis was therefore 48 095.

Most patients were diagnosed when aged between 50 and 69 years (Table 2). Twenty-one per cent of patients were <50 years and only 4% were over the age of 80 years. Each geographical region within England contributed a few thousand patients to the analyses. Tumour location was lateral for 59%, medial for 24% and central for 9%. In another 9% of patients, the tumour involved more than one quadrant (overlapping). Tumour stage was T1 (2 cm or less) for 62% of women, T2 (2–5 cm) for 33%, T3 (>5 cm) for 5% and T4 (extension to the chest wall or skin) for 1%. Most women had axillary node-negative disease (67%); the percentages of patients with node-positive disease were: one to three involved nodes 24%, four to nine involved nodes 6% and 10 + nodes 2%. Most patients were oestrogen receptor positive (87%) and HER2 negative (89%). Tumour grade was low in 18% of women, intermediate in 52% and high in 30%. Most women (82%) underwent breast-conserving surgery; the other 18% had mastectomy.

### Percentage of Patients Meeting Different Internal Mammary Chain Radiotherapy Criteria

The percentage of patients who would fulfil IMC radiotherapy criteria varied substantially between countries from 13% (6035/48 095) to 59% (28 373/48 095) (Table 3). The highest percentage of patients meeting criteria for IMC radiotherapy recommendation or consideration was for the National Comprehensive Cancer Network (NCCN) USA guidelines (59%) followed by Ireland (56%) and then Germany (32%). The UK had the lowest percentage meeting the criteria (18% and 13%).

For the UK RCR guidelines, 13% (6035/48 095) of patients fulfilled criteria for considering IMC irradiation. Nine per cent (4163/48 095) had T4 and/or N2–3 disease and 4% (1872/48 095) had one to three axillary macrometastases and a medial or central lesion and also another risk factor (age ≤ 50 years, oestrogen receptor negative, HER2 receptor positive or tumour grade 3). For the NICE guidelines, 18% (8816/48 095) of patients fulfilled criteria for considering IMC irradiation. Eight per cent (4015/48 095) had four or more positive axillary nodes and 10% (4801/48 095) had one to three positive nodes and T3/4 disease or high-grade tumours. Based on the German DEGRO guidelines, a total of 32% (15 646/48 095) would have been recommended or considered for IMC radiotherapy. Eight per cent (4015/48 095) would have had IMC radiotherapy recommended because they had more than three involved axillary nodes and in 24% (11 631/48 095) it would have been strongly considered because they had one to three involved nodes. Based on the Irish guidelines, 56% (26 846/48 095) would have been considered for IMC radiotherapy, including 33% (15 646/48 095) with node-positive disease and 23% (11 200/48 095) with node-negative disease but with inner quadrant tumours. For the USA, two guidelines were identified. Considering the NCCN guidelines, 59% (28 373/48 095) of patients would have been recommended or considered for IMC radiotherapy. IMC radiotherapy would have been recommended for 8% (4015/48 095) because they had four or more positive axillary nodes and would have been strongly considered for 24% (11 631/48 095) because they had one to three positive nodes. It would have been considered in patients who were post-mastectomy with negative axillary nodes but whose tumours were >5 cm (1%; 501/48 095) or with a central or medial location (1%; 536/48 095) and in a further 22% (10 485/48 095) who had breast-conserving surgery with negative axillary nodes but who had a central or medial lesion. It would also have been considered for patients with negative axillary nodes but whose tumours were >2 cm with other high-risk features (3%; 1205/48 095). A second clinical practice guideline (American Society of Clinical Oncology, American Society for Radiation Oncology and Society of Surgical Oncology) from the USA recommended IMC radiotherapy for patients post-mastectomy with clinical stage I and II cancers and axillary nodal involvement after neoadjuvant systemic therapy [12]. This second clinical practice guideline did not give criteria for other categories of women so it was not possible to calculate the total number of patients who would be considered for IMC radiotherapy based on this guideline.

### Sensitivity Analyses

Because data were complete for only 48 095 of the 111 729 possible patients (43%) (Figure 2), sensitivity analyses were conducted (Appendix A). To estimate the effect of missing data, analyses were conducted for each criterion for IMC radiotherapy (each row of Table A.1) based on the number of women with available information for the specific factors relevant to that particular criterion. Analyses compared the percentages of patients who would fulfil the criteria in each row based first on all women with available data for that row and second just on the 48 095 who had complete data for all variables. For example, in the RCR guideline row 1, analysis including just the 48 095 women with complete data yielded 9% of patients eligible. Analysis including all 92 239 women with available data on T and N stage (who may have been missing other data items) also yielded 9% of women eligible. Similar patterns were also seen for RCR row 2 and for the other guidelines, suggesting that our results were not materially affected by missing data.

### Analyses Using Individual Patient Data in a Single UK Centre

Data were available for 305 consecutive patients who received adjuvant breast cancer radiotherapy in a single cancer centre in South East England during July to December 2014 (Appendix B). A comparison of the percentages who would fulfil each of the criteria for IMC radiotherapy showed that the individual patient notes review, although based on much smaller numbers of patients, yielded similar patterns to patients irradiated in the whole of England during 2012–2016 (Table B.3).

## Discussion

The percentage of women undergoing radiotherapy for early breast cancer in the whole population of England during 2012–2016 who met the UK RCR criteria for IMC radiotherapy was 13%. Criteria in different national guidelines varied, leading to substantial variation in the percentages of women studied who may be considered for IMC radiotherapy in different countries, from 13 to 59%.

Our systematic search of national radiotherapy guidelines identified only six guidelines from four countries that presented criteria for ‘consideration of’ or ‘recommendation for’ IMC radiotherapy. This suggests that for many countries either there are no agreed national guidelines or that they have not been published formally in the open literature in English but may be available on local country-specific websites. One guideline from Spain (GEORM) published in 2013 [7] recommended against IMC radiotherapy altogether. However, the authors reported that they were waiting for publication of the EORTC 22922/10925 trial results, so it is likely that updated guidelines will be published soon. National guidelines from Japan and Belgium and an international guideline from the European Society of Medical Oncology referred to IMC radiotherapy but did not offer specific criteria for which patients should be considered for this treatment [5], [6], [24].

In the four countries that published IMC radiotherapy criteria, different expert panels assessed the same large studies but came to different conclusions about how to apply the evidence. For example, in the UK, where historically IMC radiotherapy has rarely been used, criteria for IMC radiotherapy included only high-risk node-positive disease [4], [19]. In Germany, the criteria included all node-positive patients [10]. In Ireland, they included some node-negative patients [13]. In the USA, where historically IMC radiotherapy was given to about 10–30% of patients, criteria for consideration of IMC radiotherapy included all node-positive patients as well as node-negative patients who had other risk factors for cancer recurrence [9]. These differences in guidelines will probably lead to substantial variations in IMC radiotherapy practice in different countries. For example, our results suggest that around a half of all patients receiving breast cancer radiotherapy would be considered for IMC radiotherapy if they were treated in the USA but not if treated in the UK (59% in the USA minus 13% in the UK = 46%).

The main limitation of this study is that it is limited to guidelines published in English. Therefore, the variation in criteria for ‘consideration of’ or ‘recommendation for’ IMC radiotherapy observed across the world may be underestimated. For example, in Denmark, a national guideline is available in Danish that recommends IMC radiotherapy for all patients with positive axillary lymph nodes and for other patients who are node-negative but who have tumours >5 cm post-mastectomy [25].

Investigating the percentage of patients meeting various IMC radiotherapy criteria can inform resource allocation. For IMC radiotherapy, contouring of the lymph node regions and organs at risk (heart, lungs and contralateral breast) is recommended [26], [27], which increases radiotherapy planning time. Radiotherapy that includes the IMC tends to increase doses to the heart and lungs compared with non-IMC radiotherapy [28], [29], which increases the relative risks of radiation-related heart disease and lung cancer [30], [31]. For each individual woman, the benefits need to be balanced against the risks. Absolute risks for individual patients are higher for patients who smoke or have cardiovascular risk factors prior to their radiotherapy. Some patients may need breathing adaptation or advanced radiotherapy techniques, e.g. volumetric modulated arc radiotherapy, to reduce heart and lung exposure [32], [33]. Implications for resource allocation will differ according to country. The RCR UK criteria for IMC radiotherapy were the most limited and were met by only 13% of patients in our study. The NICE criteria for IMC irradiation are similar to the RCR consensus [4] but differ in how many women with one to three nodes are considered. In the NICE guidelines, the location of the primary tumour within the breast is not specified as a criterion, whereas in the RCR consensus, only women with central or medial disease with one to three nodes are considered. Extending recommendations beyond the UK criteria to meet the DEGRO criteria would mean considering IMC radiotherapy for around a third of irradiated patients (32%). Extending recommendations to include other subsets of patients with node-negative disease would further increase the number of patients considered for IMC radiotherapy up to around 60%. Appreciably more resources may be needed in countries using broad criteria for IMC radiotherapy, e.g. the USA, than in countries with narrow criteria, e.g. the UK.

## Conclusions

There is substantial variability between countries in criteria for consideration of IMC radiotherapy, despite being based on the same evidence. The percentage of patients who would be considered for it varied substantially based on different national criteria. This will probably lead to large variations in practice and resource needs. This information may aid decisions regarding the feasibility of offering IMC radiotherapy to different categories of patients.

## Conflict of interest

The authors declare that there are no conflicts of interest.
