# Supplementary material for: International Variation in Criteria for Internal Mammary Chain Radiotherapy
Source: Clin Oncol (R Coll Radiol). 2019 Jul;31(7):453–61. doi: 10.1016/j.clon.2019.04.007 (PMC6575151; doi:10.1016/j.clon.2019.04.007)
Supplement: Multimedia component 1 [file mmc1.pdf]

Table A.1 Sensitivity analysis based on all 111,729 patients, including those with some missing variables.
Patients recorded as receiving adjuvant breast cancer radiotherapy in England during 2012-2016, who would fulfil criteria for internal mammary chain radiotherapy in national and international guidelines.

Clinical Guidelines	Criteria for "consideration of" and "recommended for" internal mammary chain radiotherapy	Patients meeting criteria		
		Number/total with information on factors	% based on all 111,729 patients	% based on 48,095 women with complete information (Table 3)
Royal College of Radiologists, UK (RCR)[1]				
Consider	T4 and/or N2-3 disease	8382 / 92239	9	9
Consider	1-3 axillary macrometastases and central/medial disease, who have been recommended locoregional irradiation based on risk factors*(including age and tumour biology)	1905 / 48488	4	4
			**13**	**13**
National Institute of Clinical Excellence (NICE)[2]				
Consider	≥4 positive axillary nodes	8134 / 93220	9	8
Consider	1-3 positive axillary nodes if T3/4 or high grade	9327 / 91617	10	10
			**19**	**18**
German Society of Radiation Oncology (DEGRO)[3†]				
Recommend	>3 involved axillary lymph nodes	8134 / 93220	9	8
Strongly consider	1–3 involved axillary lymph nodes	22963 / 93220	25	24
			**34**	**32**
Department of Health, Ireland (NCCP)[4]				
Consider	Positive axillary nodes	31097 / 93220	33	33
Consider	Negative axillary nodes, inner quadrant tumours	14290 / 61670	23	23
			**56**	**56**
National Comprehensive Cancer Network, USA (NCCN)[5‖‡]				
Recommend	≥4 positive axillary nodes	8134 / 93220	9	8
Strongly consider	1-3 positive axillary nodes	22963 / 93220	25	24
Consider	Negative axillary nodes and tumour >5 cm post mastectomy	1068 / 92239	1	1
Consider	Negative axillary nodes, central/medial tumour* ≤5 cm post mastectomy	677 / 61072	1	1
Consider	Negative axillary nodes, central/medial tumour post breast conserving surgery	13382 / 61670	22	22
Consider	Negative axillary nodes, lateral tumour* >2 cm with high-risk features (young age)[§]	1514 / 61072	2	3
			**60**	**59**

Table includes 6 studies in four countries. The UK included two studies[1,2.] In the USA, a second eligible guideline was identified[6] which was not included in the table. See footnote ‖.
Guidelines are ordered according to the total percentage of women fullfilling criteria.

*We used the following risk factors: age ≤50 years, oestrogen receptor negative, Her2 receptor positive and grade 3.
†The DEGRO expert panel also concluded that nodal irradiation can be discussed with node negative patients with risk factors on a case by case basis.
‖A second clinical practice guideline (American Society of Clinical Oncology, American Society for Radiation Oncology, and Society of Surgical Oncology) from the U.S.A was identified. This guideline only related to radiotherapy for patients who received mastectomy and had T1-2 breast cancer with 1-3 positive nodes so it was not included in the table.
‡ The NCCN guidelines also reported internal mammary chain radiotherapy should also be considered for patients post-mastectomy with positive margins when re-excision to negative margins is not feasible. Margin data were unavailable for patients in the listed categories.
§ We defined ≤ 50 years as young

1. The Royal College of Radiologists. Postoperative radiotherapy for breast cancer: UK consensus statements. London: The Royal College of Radiologists, 2016.
2. National Institute for Health and Care Excellence (Last updated: July 2018) Early and locally advanced breast cancer: diagnosis and treatment (NICE Clinical Guideline NG101). Available at: https://www.nice.org.uk/guidance/ng101/chapter/Recommendations#radiotherapy
3. Krug D, Baumann R, Budach W, et al. Current controversies in radiotherapy for breast cancer. Radiation Oncology. 2017 (1):25.
4. Department of Health. Diagnosis, staging and treatment of patients with breast Cancer. National Clinical Guideline No. 7. June 2015. ISSN 2009-6259
5. National Comprehensive Cancer Network (NCCN) Version 4.2017-February 7,2018. Accessed 03 March 2018 https://www.nccn.org.
6. Recht A, Comen EA, Fine RE, et al. Postmastectomy radiotherapy: an American society of clinical oncology, american society for radiation oncology, and society of surgical oncology focused guideline update. Pract Radiat Onc 2016; 6(6):e219-34

Appendix B.

Analyses repeated using individual patient data in a single UK centre

Methods

Patients included, variables and information sources

Identifiers of patients who received radiotherapy to the breast or chest wall +/- lymph nodes during 1st July 2014 to 31st December 2014 were obtained from the radiotherapy CT-planning database at the Oxford Cancer and Haematology Centre, Oxford University Hospitals NHS Foundation Trust (Fig B.1). Patients were excluded if they received radiotherapy for ductal carcinoma in situ alone (i.e. they did not have invasive cancer), if they had metastatic disease, or if radiotherapy was given for local recurrence of a previous cancer. The study was reviewed by the Research Governance Team at the Oxford University Hospitals NHS Trust.

The patient variable age was abstracted from clinical notes. Tumour variables: laterality, location, and stage were extracted both from histology reports and from radiology reports in the following order (Table B.1, B.2). Histology records were searched first. If laterality and location were missing then they were sought from radiology reports (mammogram, ultrasound and MRI). If these were still missing, radiotherapy CT-planning scans were used to identify the location of the surgical bed. For patients who received neo-adjuvant chemotherapy histology reports can only describe tumour stage after chemotherapy. For these patients information on tumour stage was sought from pre-chemotherapy investigations including mammogram, ultrasound and MRI reports. Pathological tumour characteristics were abstracted from histology reports.

Patient categorisation

Each patient was categorised according to whether she/he met criteria for consideration of IMC radiotherapy in each set of national guidelines. The number of patients in whom IMC radiotherapy would be considered or recommended in each set of national guidelines was calculated by totalling the patients who fulfilled each individual criterion (individual rows, Table B.3). The percentage of patients in whom IMC radiotherapy would be considered or recommended was calculated by dividing this number by the total number of patients (305).

Results

During July-Dec 2014, 343 patients underwent radiotherapy to the breast or chest wall +/- lymph nodes in the Oxford cancer centre (Fig B.1). 38 patients were excluded: 22 received radiotherapy for ductal carcinoma in situ only, 9 had metastatic disease, 4 were irradiated for local recurrence of a previous cancer and for 3 patients the clinical notes, histology and radiotherapy reports were unavailable. 305 patients were included. Six patients had bilateral breast cancer yielding 311 cancers for analysis.

The median age at diagnosis was 63 years (range 32-90 years) and 18% of patients were <50 years (Table B.2). The tumour location was lateral for 52%, medial for 31%, central for 16% and unknown for <1%. The tumour stage was T1 for 48%, T2 for 41%, T3 for 10% and T4 for 1%. The percentage of patients with node-positive disease was; 1-3 positive nodes: 24%, 4-9 positive nodes: 7% and 10 or more positive nodes: 3%. The percentage of patients with adverse pathological factors was: oestrogen receptor (ER) negative: 17%, HER2 receptor positive 17%, tumour grade high: 40% and lymphovascular invasion (LVI): 35%. Most patients (77%) underwent breast-conserving surgery; the other 23% had mastectomy. Comparison with the percentages of patients with these variables in the whole of England during 2012-2016 yielded similar patterns.

Percentage of patients meeting different IMC radiotherapy criteria

As in patients irradiated for breast cancer in the whole population of England, the percentage of patients fulfilling IMC radiotherapy criteria varied substantially between countries (Table B.3) and was highest for the NCCN USA guidelines (71%) followed by Ireland (55%) then Germany (34%) and the UK (Royal College of Radiologists 17% and National Institute of Clinical Excellence 24%). The ordering of guidelines according to the percentage of patients who would be recommended or considered for IMC radiotherapy was the same for patients in the single centre and in the whole of England, but with some expected differences in the absolute values of the percentages.

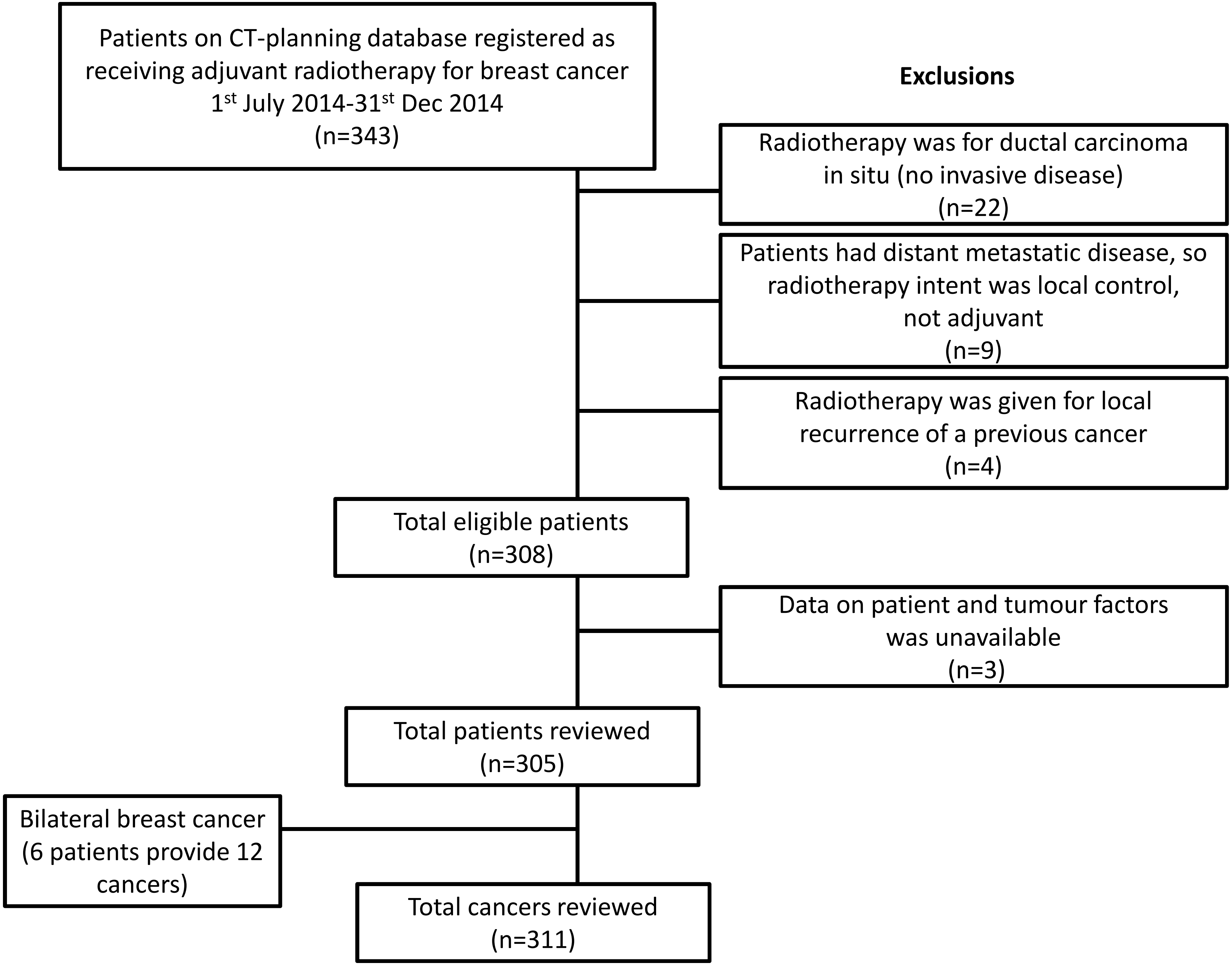

Fig B.1. Composition of study population among patients irradiated for breast cancer in a single UK centre during July-December 2014

Table B.1. Information sources used to abstract variables in 305 patients irradiated for breast cancer in a UK centre during July-December 2014

Variables	Source
**Patient**	
Age	Clinical notes or letters
Sex	Clinical notes or letters
**Tumour**	
Laterality	Histology reports
Location	Histology reports<br>Mammogram reports<br>Ultrasound reports<br>MRI reports<br>Radiotherapy CT-planning scans
Stage	Histology reports<br>Mammogram reports<br>Ultrasound reports<br>MRI reports
Pathological characteristics	
Oestrogen receptor status	Histology reports
Progesterone receptor status	Histology reports
Her2 receptor status	Histology reports
Tumour Grade	Histology reports
Lymphovascular invasion	Histology reports
**Systemic Treatment**	
Chemotherapy	Chemotherapy database
Endocrine therapy	Clinical notes or letters

Table B.2 Patient and tumour variables among 305 patients irradiated for 311 cancers in a single centre in South East England during July-December 2014.

Variable	No.	%
(a) **Patient**	No./305	
Age, years at diagnosis		
<40	12	4
40-49	43	14
50-59	72	24
60-69	97	32
70-79	61	20
80+	20	6
Sex		
Female	304	>99
Male	1	<1
(b) **Tumour**	No./311	
Breast cancer laterality		
Left	156	51
Right	143	47
Bilateral	6	2
Tumour location*		
Lateral	162	52
Central	50	16
Medial	96	31
Unknown	3	<1
Tumour stage		
T1: ≤2 cm	149	48
T2: >2-5 cm	128	41
T3: >5 cm	30	10
T4: Spread to skin/chest wall	4	1
Number of positive nodes		
0/microscopic	206	66
1-3	76	24
4-9	21	7
10 or more	8	3
Oestrogen receptor status		
Positive	259	83
Negative	52	17
Her2 receptor status		
Positive	53	17
Negative	253	81
Unknown	5	1
Tumour Grade		
Low	46	15
Intermediate	140	45
High	125	40
Lymphovascular invasion		
Positive	109	35
Negative	191	61
Unknown	11	4
(c) **Treatment**	No./311	
Type of surgery		
Mastectomy	71	23
Breast conserving	239	77
Other	1	<1

Table B.3. Percentages of patients irradiated for breast cancer in a single centre in South East England during July-December 2014, who would fulfil criteria for internal mammary chain radiotherapy in national and international guidelines, compared to patients in the whole of England.

Clinical Guidelines	Criteria for "consideration of" and "recommended for" internal mammary chain radiotherapy	Patients meeting criteria: Number/305	%	% in Public Health England data (Table 3)
Royal College of Radiologists, UK (RCR)[1]				
Consider	T4 and/or N2-3 disease	31	10	
Consider	1-3 axillary macrometastases and central/medial disease, who have been recommended locoregional irradiation based on risk factors[†] (including age and tumour biology)	22	7	
		53	**17**	**13**
National Institute of Clinical Excellence (NICE)[2]				
Consider	≥4 positive axillary nodes	29	9	
Consider	1-3 positive nodes if T3/4 or high grade	46	15	
		75	**24**	**18**
German Society of Radiation Oncology (DEGRO)[3 ǁ]				
Recommend	>3 involved axillary lymph nodes	29	9	
Strongly consider	1–3 involved axillary nodes	76	25	
		105	**34**	**32**
Department of Health, Ireland (NCCP)[4]				
Consider	Positive axillary nodes	105	34	
Consider	Negative axillary nodes, inner quadrant tumours	65	21	
		171	**55**	**56**
National Comprehensive Cancer Network, USA (NCCN)[5‡§]				
Recommend	≥4 positive axillary nodes	29	9	
Strongly consider	1-3 positive axillary nodes	76	25	
Consider	Negative axillary nodes and tumour >5 cm post mastectomy	7	2	
Consider	Negative axillary nodes, central/medial tumour ≤5 cm post mastectomy	9	3	
Consider	Negative axillary nodes, central/medial tumour post breast conserving surgery	84	28	
Consider	Negative axillary nodes, lateral tumour >2 cm with high-risk features (young age or extensive LVI[¶])	13	4	
		218	**71**	**59**

Table includes 6 studies in four countries. The UK included two studies[1,2]. In the USA, a second eligible guideline was identified[6] which was not included in the table. See footnote[‡].
Guidelines are ordered according to the total percentage of women fullfilling criteria.

[†] We used the following risk factors: age ≤50 years, oestrogen receptor negative, progesterone receptor negative, Her2 receptor positive and grade 3.
|| The DEGRO expert panel also concluded that nodal irradiation can be discussed with node negative patients with risk factors on a case by case basis.
[‡] A second clinical practice guideline (American Society of Clinical Oncology, American Society for Radiation Oncology, and Society of Surgical Oncology) from the U.S.A was identified. This guideline only related to radiotherapy for patients who received mastectomy and had T1-2 breast cancer with 1-3 positive nodes so it was not included in the table.
[§]The NCCN guidelines also reported internal mammary chain radiotherapy should also be considered for patients post-mastectomy with positive margins when re-excision to negative margins is not feasible. Twelve patients with positive margins were included in the listed categories.
¶ We defined ≤ 50 years as young and any LVI as extensive LVI because information on the extent of LVI was unavailable.

1. The Royal College of Radiologists. Postoperative radiotherapy for breast cancer: UK consensus statements. London: The Royal College of Radiologists, 2016.
2. National Institute for Health and Care Excellence (Last updated: July 2018) Early and locally advanced breast cancer: diagnosis and treatment (NICE Clinical Guideline NG101). Available at: https://www.nice.org.uk/guidance/ng101/chapter/Recommendations#radiotherapy
3. Krug D, Baumann R, Budach W, et al. Current controversies in radiotherapy for breast cancer. Radiation Oncology. 2017 (1):25.
4. Department of Health. Diagnosis, staging and treatment of patients with breast Cancer. National Clinical Guideline No. 7. June 2015. ISSN 2009-6259
5. National Comprehensive Cancer Network (NCCN) Version 4.2017-February 7,2018. Accessed 03 March 2018 https://www.nccn.org.
6. Recht A, Comen EA, Fine RE, et al. Postmastectomy radiotherapy: an American society of clinical oncology, american society for radiation oncology, and society of surgical oncology focused guideline update. Pract Radiat Onc 2016; 6(6):e219-34
